# Graphene oxide–cerium oxide nanocomposite modified gold electrode for ultrasensitive detection of chlorpyrifos pesticide

**DOI:** 10.1039/d4ra04406a

**Published:** 2024-09-02

**Authors:** Sami Ullah Khan, Waqas Khalid, Muhammad Atif, Zulqurnain Ali

**Affiliations:** a Smart Surfaces and Materials Group, Functional Materials Lab, Department of Physics, Air University PAF Complex Islamabad Pakistan waqas.khalid@au.edu.pk +92-51-9153-721

## Abstract

This research presents a novel approach for the detection of the pesticide chlorpyrifos (CLP) using a gold working electrode immobilized with a graphene oxide–cerium oxide (GO–CeO_2_) nanocomposite in a phosphate buffer (PBS) solution with a pH of 7.0. Graphene oxide (GO) was synthesized *via* a modified Hummer's method, while cerium oxide (CeO_2_) nanoparticles were prepared using a coprecipitation technique. The GO–CeO_2_ nanocomposite was synthesized *via* sonochemical methods. Structural and morphological characterization of the prepared material was conducted using X-ray diffraction (XRD) and scanning electron microscopy (SEM) coupled with energy dispersive spectroscopy (EDX). Fourier transform infrared (FTIR) spectroscopy has been conducted for the confirmation of functional group presence in the prepared materials. Cyclic voltammetry (CV) was employed to investigate the interaction between the prepared material and the analyte. Further investigations using varying scan rates (5 mV s^−1^ to 300 mV s^−1^) revealed a diffusion-controlled process at the electrode–electrolyte interface. Linear sweep voltammetry (LSV) experiments were conducted across a pH range of 5 to 9, with pH 7.0 showing enhanced response for the target pesticides in the presence of the buffer solution. Subsequent electrochemical measurements were performed at pH 7.0. Chronocoulometry was utilized to measure the effective electrode area for electrochemical interactions. Ultrasensitive square wave voltammetry (SWV) was employed for investigating the sensitivity over a concentration range of 1 fM to 100 μM and yielded the limit of detection (LOD) and limit of quantification (LOQ) as 47.7 fM and 159 fM respectively. Interference studies confirmed the selectivity of the prepared sensor, while stability and reproducibility were assessed through controlled experiments. Electrochemical impedance spectroscopy (EIS) was performed to investigate the interactions at the interface. This study provides insights into the development of selective electrochemical sensors for pesticide detection, with potential applications in environmental monitoring.

## Abstract 2

This research introduces a noval method to detect chlorpyrifos (CLP) using a gold electrode coated with a graphene oxide–cerium oxide (GO–CeO_2_) nanocomposite in a pH 7.0 phosphate buffer solution.

## Graphical abstract

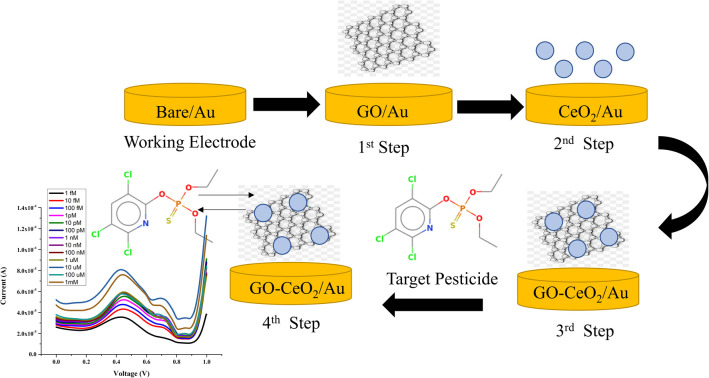

## Introduction

Pesticides serve as essential neurotoxic agents in agricultural pesticides, offering significant advantages in managing insect pests, diseases, and enhancing plant growth for improved yield and quality. However, the inefficiency of the pesticide application, where less than 0.1% reaches the intended target,^1^ necessitates excessive use for economic gains.^2^ This trend has led to widespread environmental contamination, adversely impacting soil, water, air, and non-target organisms.^3,4^ Consequently, pesticide residue contamination in agricultural produce has emerged as a global concern. Despite the associated risks, pesticides remain indispensable for pest and disease management, leading to overreliance on these chemicals.^5,6^ The classification of chlorpyrifos (CLP) as a class II moderately hazardous pesticide underscores its widespread use as a broad-spectrum organophosphate insecticide, nematicide and acaricide.^7,8^ Numerous studies have detected CLP residue in various crops,^9–12^ highlighting the urgency for rapid and effective detection methods in both plants and drinking water to mitigate health risks. The World Health Organization's classification and prevalence of CLP residue emphasizes the critical need for urgent research in this area.

Organophosphate (OP) insecticides have become increasingly common as a means of raising agricultural yields.^13^ They are extensively employed in pre- and post-harvest interventions to manage several ailments affecting a variety of fruits and vegetables.^14,15^ The environment and public health are seriously at risk due to their widespread use. Even though they are categorized as persistent pesticides with harmful effects on the environment, they are nevertheless widely utilized in many nations, which has resulted in significant contamination of food samples and ground water. OP pesticides are strong inhibitors of acetyl cholinesterase (AChE) that can result in severe cholinergic toxicity when applied topically, inhaled, or consumed.^16^ The European Union (EU) states that in drinking water and food originating from plants, the maximum allowable quantities of pesticides and the compounds linked to them are 0.1 μg L^−1^ and 0.05 mg kg^−1^, respectively (EEC, 1980).

Gas chromatography (GC), gas chromatography-mass spectrometry (GC-MS), high performance liquid chromatography (HPLC), ultra-high performance liquid chromatography-mass spectrometry (UPLCMS), and immunosensors are widely utilized for pesticide residue detection.^17–20^ However, these methods are hindered by intricate pretreatment procedures, time-consuming analyses, and high operational costs, especially when handling large sample volumes in agricultural processing and monitoring industries. Additionally, the use of various chemical reagents during analysis poses environmental risks.^21^ In contrast electrochemical techniques have gained prominence in recent decades due to their user-friendly operation, rapid on-site detection capabilities, cost effectiveness and reproducibility. These methods offer a practical alternative for pesticide screening, enabling timely and efficient analysis without the need of extensive sample preparation or hazardous chemicals.^22,23^ Electrochemical sensors with their simplicity and sensitivity, present a promising avenue for enhancing pesticide detection efficiency while minimizing environmental impact.

W. S. T. Tun *et al.*^24^ investigated the use of cellulose nanofibers and graphene oxide on screen printed electrode for the sensitive and selective detection of OPs, with chlorpyrifos as the model compound using acetylcholinesterase. The sensitivity of the sensor was assessed using square wave voltammetry, yielding a detection limit (LOD) of 2.2 nM and a quantification limit LOQ of 73 nM. H. Wang *et al.*^25^ developed 2D/2D Z-scheme β-Bi_2_O_3_/g-C_3_N_4_ heterojunctions for chlorpyrifos detection where the composite improved the electron detection as confirmed by photoluminescence and electrochemical impedance spectroscopy. This method achieved and LOD of 0.03 ng mL^−1^ for chlorpyrifos. Several other nonenzymatic sensors for pesticides have been reported.^26–29^ For instance, A. Kumaravel *et al.*^30^ created an electrochemical sensor by immobilizing nano TiO_2_ and cellulose acetate on a glassy carbon electrode (GCE) to detect chlorpyrifos. Using cyclic voltammetry, differential pulse voltammetry and amperometry they observed the reduction peak at −1.55 V linked to the electroreduction of the pyridine ring's C=N bond. This sensor achieved an LOD of 4.4 μM and LOQ of 14.7 μM. J. N. Nirmala *et al.*^31^ modified a GCE with stearic acid for detecting parathion and methyl parathion. Cyclic voltammetry indicated a diffusion-controlled mechanism, with a 20% current increase compared to the bare electrode. Differential pulse voltammetry showed an LOD of 0.02 μM for parathion and 0.6583 μM for methyl parathion.

CuO–TiO_2_ hybrid composite, synthesized by Tian *et al.*,^32^ serves as effective electrode modification for methyl parathion (MP) detection without enzymatic assistance. The interaction between MP and CuO nanostructures suppressed the anodic current peak, facilitating MP detection, as evidenced by the differential pulse voltammetry (DPV) analysis yielding a low detection limit of 1.21 ppb with minimal interference. Inspired by these findings, researchers explored the combination of CuO nanoparticles and two-dimensional materials to enhance sensor performance and stability. Soomro *et al.*^33^ introduced and electrochemical sensor for malathion detection, using hydrothermally synthesized CuO nanostructures and amino acids as bio-templates. The strong affinity between CuO and malathion inhibits the anodic peak current, resulting in efficient detection of organophosphates. This sensor exhibited remarkable stability, and ultra-low limit of detection (LOD) of 0.1 nM. Fu *et al.*^34^ pioneered an electrochemical sensor employing silica/exfoliated graphene composite films for MP determination. The synergistic interaction between nano-silica and graphene significantly improved the sensor's current response compared to bare glassy carbon electrode (GCE). Similarly, Song *et al.*^35^ developed a sensor based on TiO_2_ nanoparticles and graphene composite, demonstrating excellent electrocatalytic activity and sensitivity towards MP detection, attributed to graphene's rapid electron transport and TiO_2_ nanoparticles' large surface area. Additionally, Karimian *et al.*^36^ proposed a metal–organic framework (MOF)-based sensing layer functionalized with TiO_2_–graphene oxide to address challenges associated with MOF-based sensors. This sensor exhibited a low LOD of 0.2 and 1.0 nM for propoxur (PXN) and chlorpyrifos (CLP), respectively, effectively monitoring residues in vegetables and water samples. In the above presented literature, different strategies have been opted to detect the chlorpyrifos in the lower concentrations. But the limit of detection achieved for the organophosphorus pesticide ranges from μM to nM.

In this study, we modified the gold (Au) electrode with GO, CeO_2_ and GO–CeO_2_ nanocomposites individually. Structural and morphological analyses were conducted using XRD, SEM and EDX respectively. The modified electrode served as a sensing platform in a conventional electrochemical cell with Ag/AgCl reference electrode and platinum counter electrode. Cyclic voltammetry investigated the incorporation of 1 mM CLP in PBS buffer (pH 7.0), while pH optimization was achieved through LSV (0–1 V). Chronocoulometry assessed the effective area, crucial for detection. SWV evaluated sensor's sensitivity. Controlled experiments examined interference, repeatability, reproducibility, and stability demonstrating the sensor's excellent performance. EIS probed electrode–analyte interactions, contributing to comprehensive understanding. In present work, the strategy has been adopted to use the synergistic effect of both the cerium oxide and that of graphene oxide in order to achieve the ultra-lower limit of detection because of the enhanced electrocatalytic activity of the cerium oxide and high surface area, exceptional conductivity and narrow band gap which facilitates the flow of electrons from graphene oxide, the combine effect of both materials in the form of composite achieved the limit of detection up to femto molar range.

## Materials and methods

The chemicals and reagents utilized in this study were of high purity and sourced from reputable suppliers. Carbendazim (97% purity), carbaryl Pestanal (analytical standard), and chlorpyrifos Pestanal™ (analytical standard) were procured from Sigma-Aldrich. Cerium nitrate hexahydrate Ce(NO_3_)_3_·6H_2_O with a purity of 99%, graphite powder (99% purity), and potassium permanganate (KMnO_4_) with a purity of 99.5% were also obtained. Additionally, hydrogen peroxide (H_2_O_2_) with a concentration of 30%, sodium hydroxide (NaOH) with a purity of 98.5% and sulfuric acid (H_2_SO_4_) with a concentration of 98.5% were sourced. Sodium chloride (NaCl) and potassium chloride (KCl) with purities of 99% and 99.5% respectively, were acquired. Furthermore, potassium hydrogen phosphate (KH_2_PO_4_) and sodium hydrogen phosphate (Na_2_HPO_4_) with purities of 98% were purchased from Sigma-Aldrich. No further modifications were necessary for their use. The pH of the solutions was adjusted by the addition of hydrochloric acid (HCl) and sodium hydroxide (NaOH) to deionized water.

The synthesis of materials was conducted according to established protocols with some modifications. Graphene oxide (GO) was synthesized using a modified Hummer's method.^37^ Initially 1 g of graphite was combined with 25 mL of H_2_SO_4_ in an ice bath. Subsequently, 3 g of potassium permanganate (KMnO_4_) was slowly added with continuous stirring at temperatures below 20 °C for three hours. Following this, 50 mL of deionized water was added dropwise, ensuring the temperature remains below 50 °C. The resulting solution transitioned to a deep brown color, indicating GO formation. To complete oxidation, 100 mL of water and 5 mL of hydrogen peroxide were added successively. The product was rinsed with deionized water and ethanol, followed by drying at 60 °C.

CeO_2_ nanoparticles were synthesized *via* co-precipitation. A 0.1 M precursor solution was prepared by dissolving cerium nitrate hexahydrate (Ce(NO_3_)_3_·6H_2_O) in 100 mL of deionized water, then heated to 75–80 °C. Sodium hydroxide was dissolved in deionized water to create a 5 M solution. After 4 hours at 80 °C, the sample was washed, dried, and annealed at 450 °C.

Subsequent experiments involved dispersing 25 mg of GO in 50 mL of deionized water, followed by sonication. A 0.5 M Ce(NO_3_)_3_·6H_2_O solution and a 5 M NaOH solution were prepared and added subsequently. After sonication and washing steps, drying occurred at 100 °C for 8 hours and then annealing was done at 400 °C for 3 hours.

In this investigation, a 1 cm × 0.6 cm Au electrode was employed as the working electrode in electrochemical cell for CLP detection. The bare Au electrode underwent ultrasonic washing in sequential steps: first in deionized water, then in ethanol, and finally in acetone. Subsequently, it was air dried at room temperature. The active material was combined with PVDF at a ratio 4 : 1 in the presence of NMP (1-methyl-2-pyrrolidinone) and homogenized for 30 minutes to produce a slurry. The amount of slurry (15 μL) was then drop-cast onto the surface of the cleaned Au electrode.^38^ Following deposition, the Au electrode was dried under a standard bulb lamp for one hour. The resulting electrode configuration is denoted as GO–CeO_2_/Au.

The electrochemical experiments utilized the Gamry Reference 3000 potentiostat/galvanostat/ZRA, operating with a conventional three-electrode setup. The reference electrode employed was Ag/AgCl, while a platinum wire served as a counter electrode to complete the electrical circuit. X-ray diffraction (XRD) analysis was conducted using Shimadzu XRD-6000 instrument. For imaging and elemental analysis, a field emission scanning electron microscope (FESEM) equipped with energy-dispersive X-ray spectroscopy (EDX) capabilities was utilized, specifically the Tescan Maia 3 model. Fourier transform infrared spectroscopy (FTIR) was performed by ATR Alpha Bruker.

## Results and discussion

**Fig. 1:**
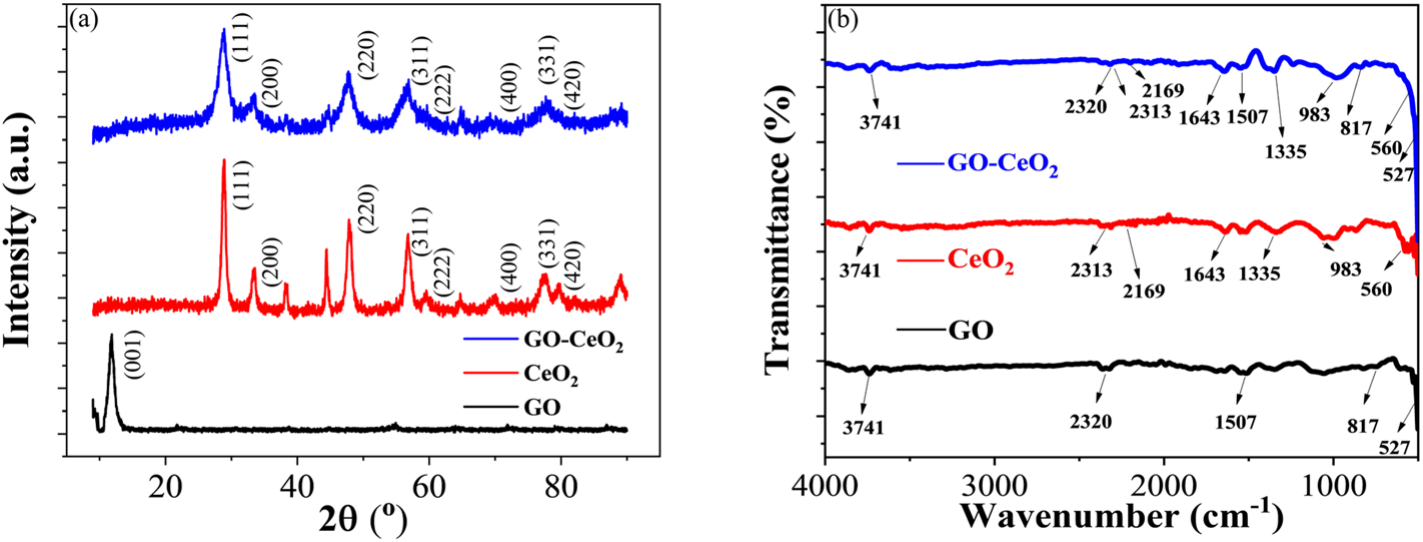
(a) XRD spectra of GO (black curve), CeO_2_ (red curve) and GO–CeO_2_ (blue curve). (b) FTIR spectra of GO (black curve), CeO_2_ (red curve) and GO–CeO_2_ (blue curve).

The X-ray diffraction (XRD) analysis was conducted to characterize the structural properties of the materials under study, including graphene oxide (GO), cerium oxide (CeO_2_) and graphene oxide–cerium oxide (GO–CeO_2_) nanocomposite. As illustrated in Fig. 1(a), GO exhibited a diffraction peak centered at 2*θ* = 11.6°, indicative of its layered structure with a *d*-spacing of 7.5 Å. The presence of oxygen functional groups and water molecules within the carbon layer structure contributed to the increased interlayer spacing.^39^ The XRD of CeO_2_ (red curve) closely matched the literature values (JCPDS no. 81-0792), with distinct peaks corresponding to the face-centered cubic phase of CeO_2_ crystal at various *θ* values. In the XRD pattern of GO–CeO_2_ nanocomposite (blue curve), the suppression of the graphene oxide peak indicated the successful incorporation of CeO_2_ in the GO matrix. The distortion in the layered structure of GO due to higher order crystallinity of CeO_2_ confirmed the effective formation of the nanocomposite. The mean crystallite size of the nanocomposite was determined to be 12 nm, while CeO_2_ nanoparticles exhibited a crystallite size of 15.9 nm, calculated using the Scherrer formula.

Fig. 1(b) displayed the FTIR spectra of GO, CeO_2_ and GO–CeO_2_ nanocomposites. The IR spectrum of GO is displayed by the black spectral line. The broad absorption band, located at 3741 cm^−1^, represents the hydroxyl moieties' –OH stretching vibrational mode on the GO matrix's surface. Bending vibrations of carbo–hydroxyl (–C–OH) of carboxylic groups and bending vibration of –C=C– cause the peak that is located at wavenumbers of 1507 cm^−1^ and 817 cm^−1^, respectively. The red line illustrates the infrared spectrum of CeO_2_, which has absorption edges at wavenumbers 983 cm^−1^ and 560 cm^−1^, respectively. These correspond to the symmetric stretching vibration of the Ce–O–Ce and the stretching of the Ce–O bands in the chains. However, the IR spectrum of GO–CeO_2_ contains absorption bands at wavenumbers 3741 cm^−1^, 1643 cm^−1^, 1335 cm^−1^, 983 cm^−1^, and 817 cm^−1^. These correspond to the stretching vibration of carbon and epoxide groups and the –OH stretching, –C=C– stretching, –C–H bending, and –OH bending of carboxylic groups, respectively. Conversely, the peaks seen at lower wavenumbers, 560 cm^−1^ and 527 cm^−1^, are associated with the stretching vibrations of Ce–O and Ce–O–Ce. It therefore validates the effective insertion of CeO_2_ nanoparticles onto the GO matrix.

**Fig. 2:**
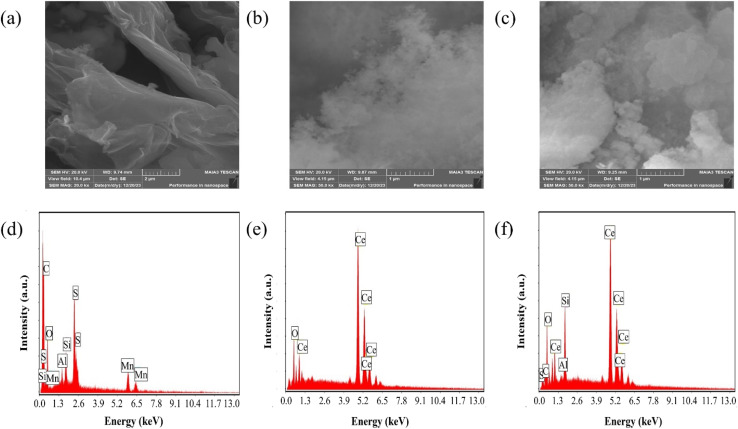
(a–c) SEM micrographs of GO, CeO_2_ and GO–CeO_2_ respectively. (d–f) Elemental analyses of GO, CeO_2_ and GO–CeO_2_ respectively.

The scanning electron microscopy (SEM) analysis, along with energy dispersive X-ray spectroscopy (EDX), provided insights into the morphology and composition of the materials under investigation. SEM images depicted in Fig. 2(a)–(c) revealed key features of GO, CeO_2_ particles and their composite system. Fig. 2(a) exhibited a uniform GO sheet with distinct layers and noticeable wrinkles, suggesting a structured morphology. CeO_2_ particles, as observed in Fig. 2(b), indicate a narrow and seemingly homogeneous size distribution. CeO_2_ particle concentration increases on the GO sheet as displayed in Fig. 2(c).

EDX analysis shown in Fig. 2(d)–(f), confirmed the elemental composition of the materials. GO demonstrated an oxygen content of 43 atom%, with a carbon to oxygen atomic ratio of 1.24, suggesting a higher oxygen content than the empirical formula proposed by Boehm *et al.*^40^ CeO_2_ nanoparticles revealed the presence of oxygen and cerium atoms without any contaminants or surfactants, as indicated by Fig. 2(e). Furthermore, EDX analyses confirmed the elemental components (cerium, oxygen and carbon) in the nanocomposite validating its composition.

Electrochemical measurements were conducted using various techniques to assess the performance of the modified electrode GO–CeO_2_/Au and its interaction with the analyte. Cyclic voltammetry (CV) explored the electrochemical behavior within a potential range of 0–1 V. Chronocoulometry (CC) determined the effective area with a potential range of −0.2 to +0.6 V. Square wave voltammetry (SWV) was employed to assess the sensitivity and limit of detection, operating within the 0–1 V potential range, with a pulse amplitude of 25 mV and integration period of 0.02 s. Electrochemical impedance spectroscopy (EIS) investigated the interfacial properties over a frequency range from 0.1 to 10^5^ Hz, applying an AC voltage of 500 mV. Additionally, linear sweep voltammetry (LSV) optimized pH conditions within the 0–1 V potential range against Ag/AgCl, utilizing a scan rate of 50 mV s^−1^.

**Fig. 3:**
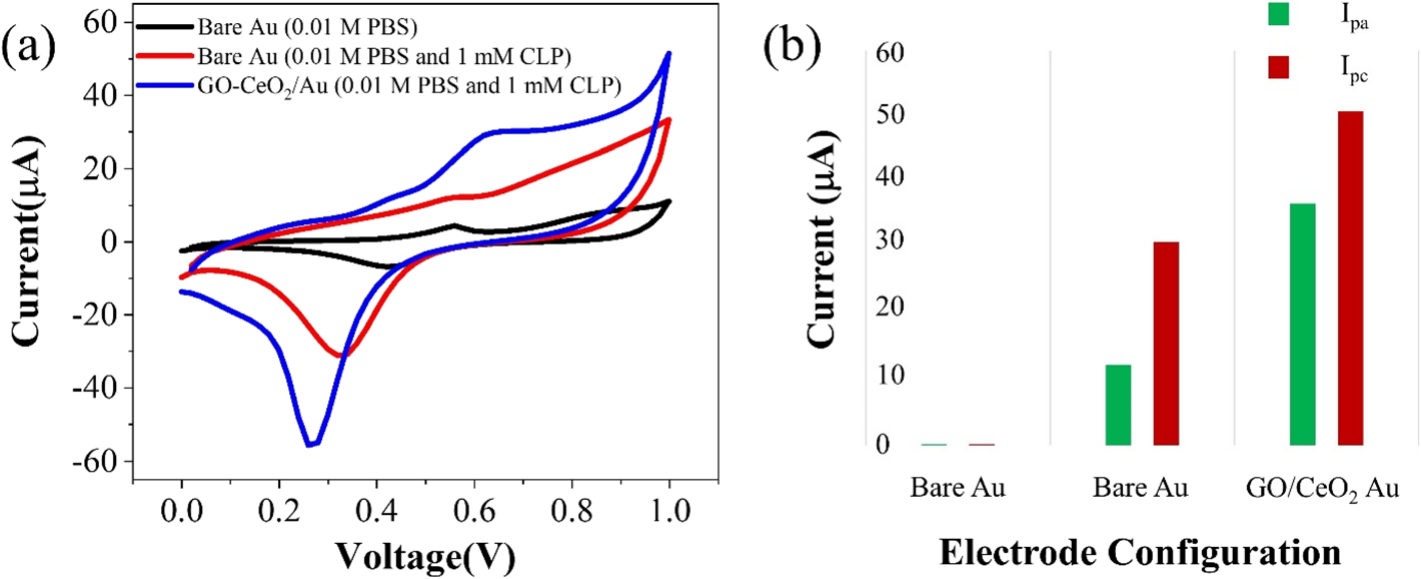
(a) Cyclic voltammetry response of Au and GO–CeO_2_/Au electrode with and without CLP. (b) Peak cathodic and peak anodic current responses for Au and GO–CeO_2_/Au electrodes.

The electrochemical behavior of the developed sensor is depicted in Fig. 3(a). The black trace represents the response on the bare Au electrode in a supporting buffer solution (PBS, pH = 7.0), showing baseline behavior. An enhanced signal (red trace) exhibits pronounced oxidation behavior and a distinct reduction peak at 320 mV upon the addition of 1 mM chlorpyrifos (CLP) analyte. Upon introduction of the modified sensor surface into the electrochemical cell with the analyte, an increased oxidative signal (blue trace) at 620 mV and prominent reduction signal at 230 mV are observed. This indicates a significant catalytic response of GO–CeO_2_ nanocomposite with CLP. Previous studies have attributed this peak to electroreduction of the pyridine's ring C=N *via* a 2e^−^ transfer process in chlorpyrifos. The presence of three chlorine atoms causes electrons delocalization at the −C=N− bond, leading to increased reduction at lower negative potentials.^41,42^

When employing only GO as the immobilizing material onto the surface of the working electrode, no discernible redox behavior is observed. Instead, only capacitive response is evident. Conversely, when CeO_2_ was utilized, clear redox behavior is observed, however the reduction peak in the case of GO–CeO_2_ nanocomposite is still more pronounced owing to the synergistic effect of the composite.

**Fig. 4:**
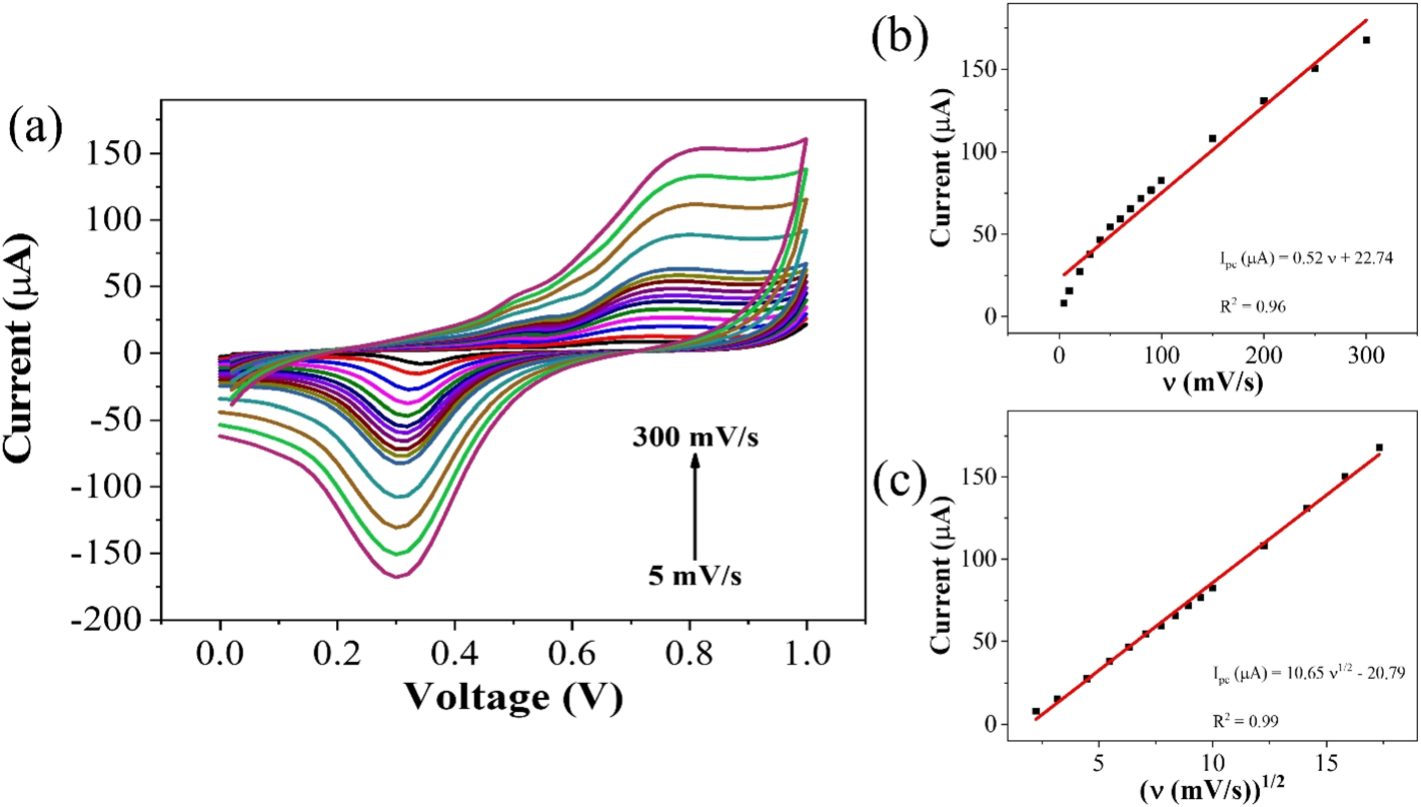
(a) Scan rate dependent response for CLP at GO–CeO_2_/Au electrode (b) plot of peak current *vs. ν* (c) plot of peak current *vs. ν*^1/2^.

To elucidate the reaction kinetics at the interface, a scan rate study was conducted over a range of 5–300 mV s^−1^ within the potential range of 0–1 V. Analysis of the voltammograms revealed a rightward shift in the anodic peak, while the cathodic peak initially shifted leftward before moving rightward. The observed behavior suggests a diffusion-controlled reaction occurring at or near the electric double layer (EDL). This interference is supported by the high correlation coefficient (*R*^2^) of 0.99 for plots of peak cathodic current (*i*_pc_) against the square root of scan rate, and 0.97 for the plots of *i*_pc_ against the scan rate itself (Fig. 4). The linear fit equation for the cathodic peak current is expressed as follows:*i*_pc_ (μA) = −(4.95 ± 0.04)*v*^1/2^ + 6.72 ± 0.44, (*R*^2^ = 0.99)

**Fig. 5:**
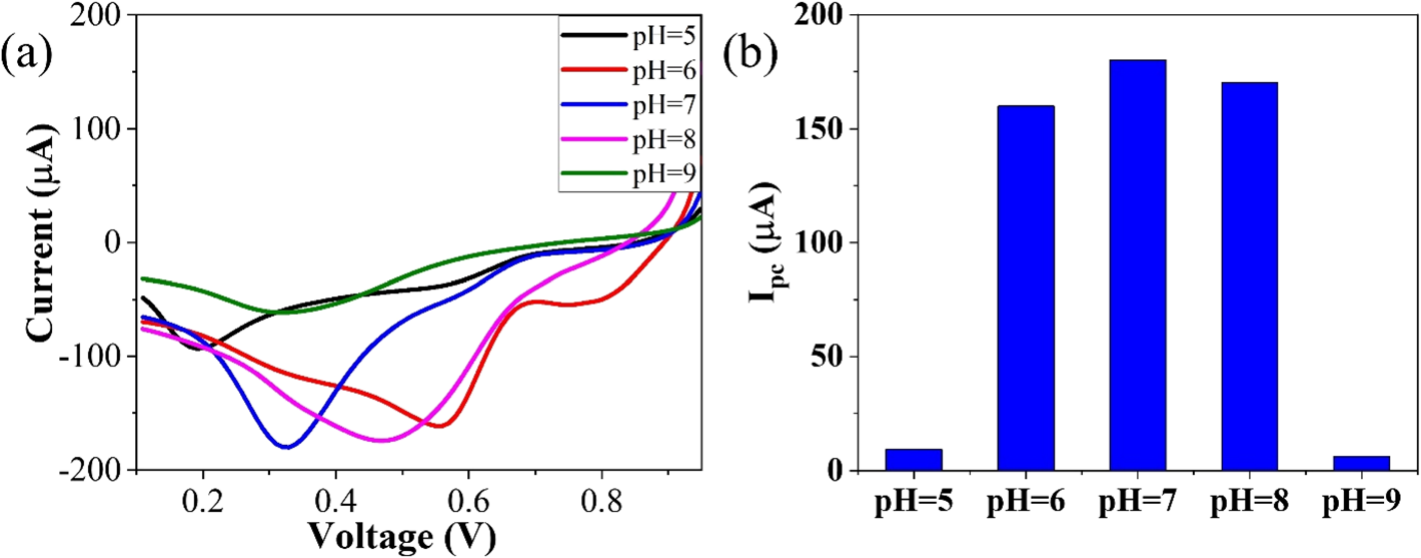
Optimization of reduction peak signals of 1 mM CLP in the presence of 0.01 M PBS.

To optimize the pH, linear sweep voltammetry (LSV) was conducted using the GO–CeO_2_/Au modified electrode within a potential range of 0–1 V in the presence of phosphate buffer saline (PBS) with a concentration of 0.01 M. The prepared solution containing the analyte CLP at a concentration of 1 mM was tested at pH values ranging from 5 to 9. The voltammograms obtained at different pH values were analyzed, and it was observed that at pH 7.0, the signal exhibited optimal characteristics, as depicted in Fig. 5. The bar graph accompanying the voltammograms illustrated the signal amplitude at each pH level. Based on these findings, a pH of 7.0 was selected as the optimal condition for the subsequent electrochemical investigations, providing consistent and reliable results for further analysis and experimentation, facilitating accurate assessment of GO–CeO_2_/Au modified electrode's performance in detecting CLP.

**Fig. 6:**
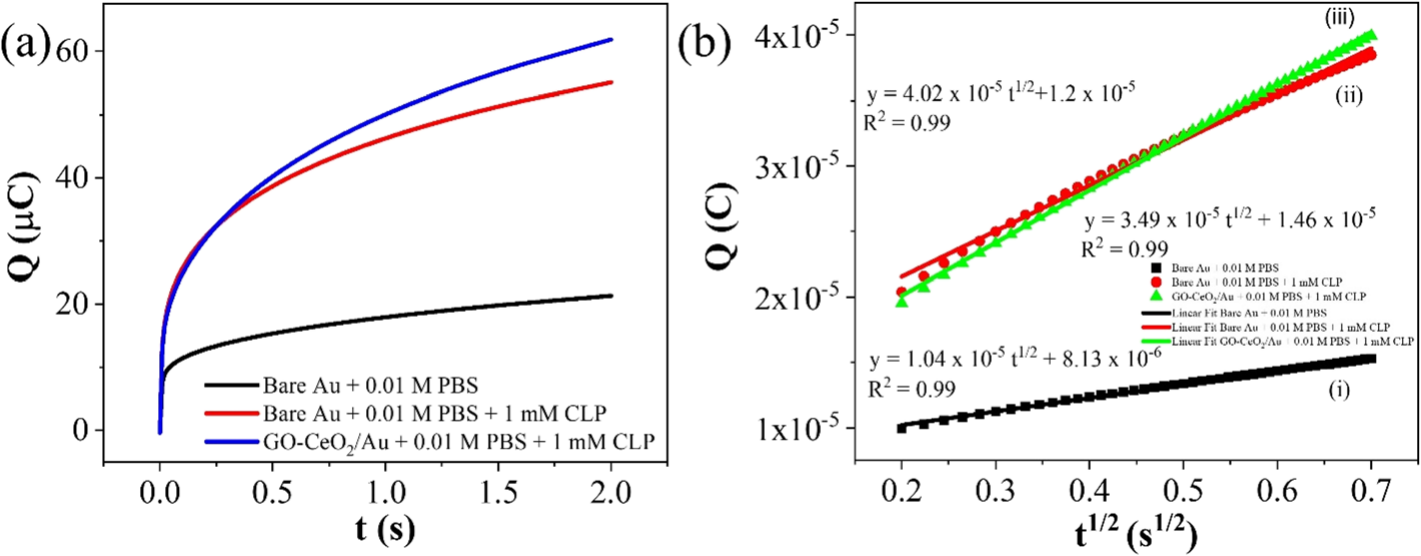
(a) Plots of *Q*–*t* curves (b) (i) *Q*–*t*^1/2^ curves for bare Au in 0.01 M PBS (ii) *Q*–*t*^1/2^ curves for bare Au in 0.01 M PBS + 1 mM CLP (iii) *Q*–*t*^1/2^ curves for GO–CeO_2_/Au in 0.01 M PBS + 1 mM CLP.

**Fig. 7:**
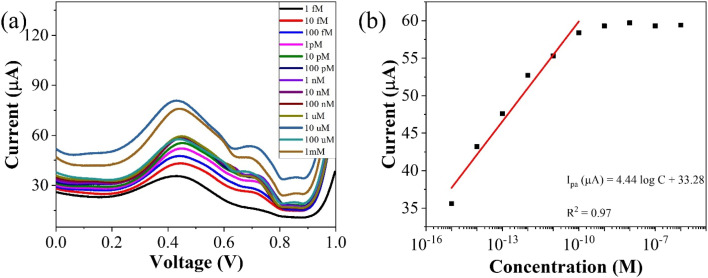
(a) Concentration profiling using SWV (b) corresponding plot of peak currents *vs.* concentration of the analyte.

The electrochemical method of chronocoulometry (CC) was employed to measure both capacitive and coulombic charge (*Q*_ad_) over time, developed at the electric double layer interface between the electrode and analyte. This technique is instrumental in determining both the effective area of the electrode and the surface charge (*Γ*) of absorbed molecules. The comparison of electrode effective areas is illustrated in Fig. 7(a). In the study, the calculated surface area of bare Au in 0.01 M PBS was found to be 0.00964 cm^2^, as shown in Fig. 7(b) (black trace). Conversely, in the presence of 0.01 M PBS (pH = 7.0) along with 1 mM CLP, the calculated area increased to 0.01658 cm^2^, depicted in Fig. 6(b) (red trace). Furthermore, when Au was immobilized with GO–CeO_2_, the effective area was further augmented to 0.02155 cm^2^ as illustrated in Fig. 6(c) (green trace). These measurements were obtained by analyzing the slope of the linear fitted curve correlating charge (*Q*) and time (*t*^1/2^), utilizing the Anson equation for the diffusion-controlled process.
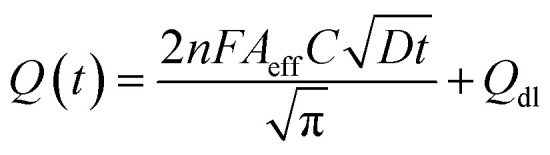
where *Q*(*t*) is the charge as function of time, *n* is the electron transfer, *F* is the Faraday's constant, *C* is the concentration of [Fe(CN)_6_]^3−/4−^, and *D* is the diffusion coefficient and *Q*_dl_ is the double layer capacitance respectively. This method provides valuable insight into the electrochemical behavior of the system and aids in understanding the kinetics of charge transfer process at the electrode surface, essential for optimizing the performance of electrochemical devices.

To assess the operational range of the prepared sensor, square wave voltammetry (SWV) was conducted across a concentration spectrum spanning from 1 fM to 1 mM. SWV is a highly effective technique utilized for both for qualitative and quantitative determination of the analyte, even when present at trace level in the solution. The methodology involves the imposition of a symmetric square wave potential onto a base staircase potential applied to the working electrode. Each complete square wave cycle corresponds temporally to one step within the staircase waveform. Within this cycle, the current is sampled twice: once at the concentration of the forward pulse (*I*_f_), and once at the termination of reverse pulse (*I*_r_). This process engenders square wave modulation, wherein reverse pulses prompt the inverse reaction of any products engendered by the preceding forward pulse. Plotting the net current (*I*_f_ − *I*_r_) against the base staircase potential yields a voltammogram, typically exhibiting a peak-shaped morphology symmetrically aligned about the half wave potential.

**Fig. 8:**
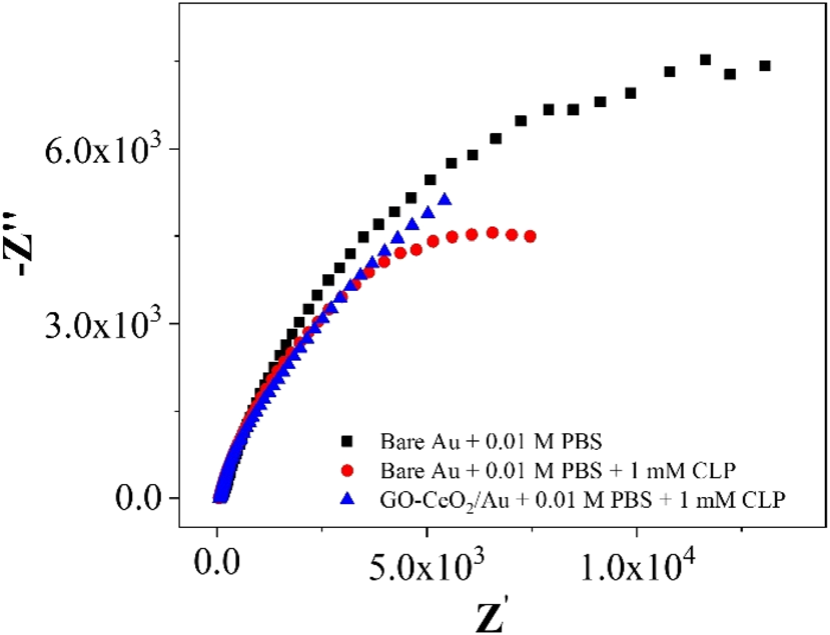
Nyquist plots of Au in presence of 0.01 M PBS and AU and GO–CeO_2_/Au in the presence of 0.01 M PBS and 1 mM CLP.

The benefits of employing the SWV over the differential pulse voltammetry (DPV) are manifold: firstly, the SWV rapid scan rate facilitates expedited analysis, minimizes the consumption of the electroactive species *vis-à-vis* a DPV, and mitigates issues associated with electrode surface blocking. Secondly, SWV boasts enhanced sensitivity compared to DPV, owing to its incorporation of reverse current considerations. Finally, SWV exhibits superior capacity for discerning background signals, particularly those stemming from oxygen reduction process. The results of the square wave voltammogram illustrates the anodic peak response of the targeted analyte, as depicted in Fig. 8(a), while the ensuing linear correlation is discernible in Fig. 8(b).

The linear fit equation is represented as follows,*i*_pa_ (μA) = (3.96 ± 0.21)log *x* + 97.41 ± 2.22, *R*^2^ = 0.97

The slope of this curve (*m*) for the blank solution and the standard deviation (*s*) can be used for the estimation of limit of detection as well as for limit of quantification as given below,
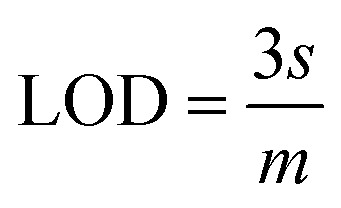

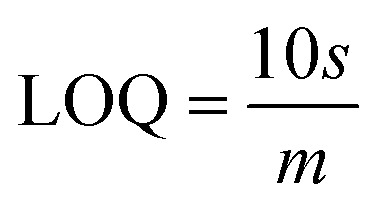


By using the above relations, we have estimated the LOD as 47.7 fM and LOQ as 159 fM respectively.

In the investigations of interfacial interactions between the modified working electrode surface and the target analyte, electrochemical impedance spectroscopy was conducted over a frequency range spanning from 0.1 to 10^5^ Hz. Experimental conditions include a DC voltage 0 V *vs. E*_ref_ and an AC voltage of 500 mV RMS, with an initial delay of 10 s. EIS spectra depicted the response of the bare Au electrode in the presence of 0.01 M PBS pH = 7.0 (black trace), the response of bare Au electrode in the presence of 0.01 M PBS pH = 7.0 and 1 mM CLP (red trace), and the response of the modified electrode (GO–CeO_2_/Au) in the presence buffer solution of 0.01 M PBS pH = 7.0 and analyte concentration 1 mM CLP (blue trace). Comparison of various metal-oxide composites with graphene oxide (GO) for pesticide detection with the current study is presented in Table 1.

**Table 1:** Comparison of different metal-oxide frameworks with GO for the detection of pesticides with current study

Material	Electrochemical technique used	Analyte	LOD	Reference
CuO-NPs/3DGR	DPV	Malathion	0.01 × 10^−9^ M	43
TiO_2_/graphene/GCE	CV, LSV	Methyl parathion	1.00 × 10^−9^ M	44
CoTCPP/Co_3_O_4_/GO	DPV	Methyl parathion	1.10 × 10^−8^ M	45
Porphyrin/Co_3_O_4_/graphene	DPV	Methyl parathion	1.10 × 10^−8^ M	46
CuO-NPs/3DGR	DPV	Malathion	0.01 × 10^−9^ M	47
Au–ZrO_2_–GNs/GCE	SWV	Methyl parathion	3.79 × 10^−9^ M	48
Au–Pd/rGO/CPE	SWASV	Parathion	8.00 × 10^−9^ M	49
TiO_2_/CA/GCE	DPV	Chlorpyrifos	3.50 × 10^−6^ M	50
CNFs/GO/CS–GO/AChE/SPCE	SWV	Chlorpyrifos	3.79 × 10^−9^ M	51
GO–CeO_2_/Au	SWV	Chlorpyrifos	47.7 × 10^−15^ M	This work

**Fig. 9:**
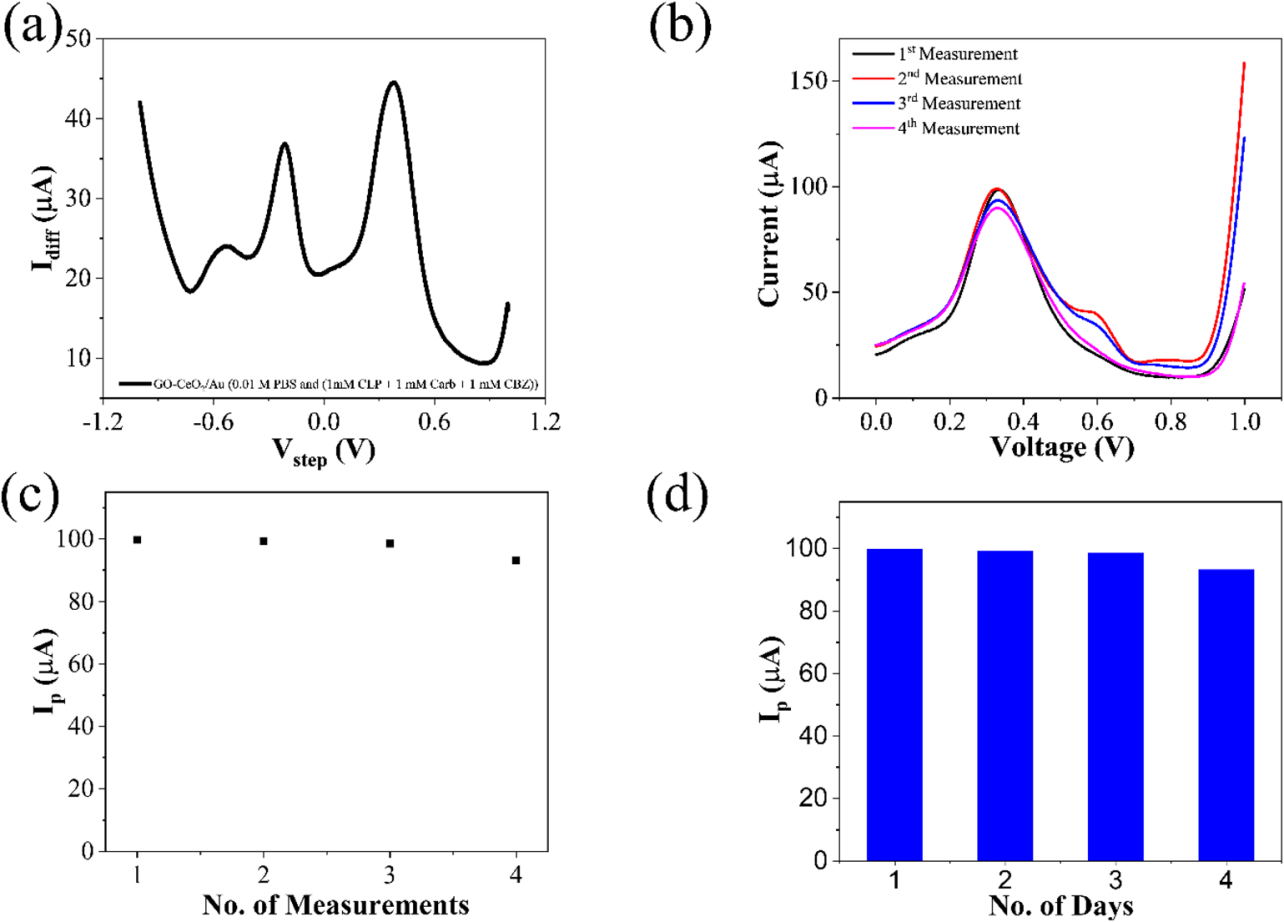
(a) Interference study of CLP in the presence of pesticides: carbaryl (CARB) and carbendazim (CBZ) (b) repeatability of the prepared sensor (c) reproducibility over several measurements and (d) stability of the prepared sensor over a period of four days.

The interference experiment was conducted by mixing three pesticides namely carbendazim, carbaryl along with the target pesticide chlorpyrifos having molarities (1 mM each) under the optimized conditions by using the square wave voltammetry in the potential range from −1 to +1 V *vs.* Ag/AgCl in the presence of 0.01 M PBS (pH = 7.0). Three distinguished peaks at −531 mV for carbendazim, −212 mV for carbaryl and 381 mV for chlorpyrifos showed the interference capability of the prepared sensor. While the enhanced peak showed the selectivity of the prepared sensor towards target pesticide chlorpyrifos in (Fig. 9(a)). Repeatability assessment involved four consecutive SWV measurements within the optimized potential window, demonstrating close alignment of all four reduction current peaks with previous measurements, indicating significant repeatability (Fig. 9(b)). Reproducibility was evaluated using for different electrodes under optimized parameters, showcasing closely matched responses (Fig. 9(c)), affirming the sensor's practical utility. Stability assessment involved monitoring the voltametric response of the same electrode over four days, with deviations from ideal behavior measured as changes in current. Corresponding reduction peaks are depicted in bar graphs (Fig. 9(d)), showing good agreement with initial measurements.

Table 2 represents the comparison of the developed systems for the detection of chlorpyrifos, which also included the electrochemical techniques employed, optimized value of pH along with buffer. Linear range of the prepared systems and limit of detection for the analyte under consideration.

**Table 2:** Comparison of different studies for the detection of chlorpyrifos pesticides with current study

Electrode/modifiers	Quantification method	pH, electrolyte	Linear range	Detection limit	Ref.
AChE/Pin5COOH/Fe_3_O_4_NP/GCE	AMP	pH 7.0 phosphate buffer	1.5 to 70 nM	9.1 nM	52
AChE/ZrO_2_/rGO	AMP	pH 7.0 phosphate buffer	10^−9^ to 10^−4^ M	10^−13^ M	53
CuO NFs-SWCNTs/GCE	DPV	pH 7.0 phosphate buffer	0.1 to 150 ng L^−1^	70 pg mL^−1^	54
NA/AuNPs/rGO–NH_2_/AChE/GCE	CV	pH 8.0 phosphate buffer	0.021 to 0.122 mg mL^−1^	14 ng mL^−1^	55
FTO–AuNPs–chl–Ab	DPV	pH 7.5 phosphate buffer	1 fM to 1 μM	10 fM	56
GO–CeO_2_/Au	SWV	pH 7.0 phosphate buffer	1 fM to 100 nM	47.7 fM	Present work

## Conclusion

The successful synthesis of a GO–CeO_2_ nanocomposite *via* sonochemical method is highlighted, with SEM images confirming the uniform distribution of CeO_2_ nanoparticles on graphene oxide sheets. The modified electrode exhibited excellent electrocatalytic activity for the detection of chlorpyrifos (CLP) in the presence of carbendazim and carbaryl in 0.01 M PBS (pH = 7.0). Graphene oxide sheets effectively prevented CeO_2_ nanoparticles agglomeration, enhancing sensitivity and selectivity for CLP detection. Scan rate studies indicate a diffusion-controlled mechanism at the interface, while chronocoulometry probed the actual electrochemical area involved. All measurements were conducted at pH 7.0, with ultra-sensitive square wave voltammetry revealing a limit of detection (LOD) and limit of quantification (LOQ) of 47.7 fM and 159 fM, respectively. Interface studies, repeatability, reproducibility, and stability assessments confirmed the sensor's practical applicability. Electrochemical impedance spectroscopy provided additional insights into interface interactions. The study contributes valuable knowledge to the development of highly sensitive selective electrochemical sensors for pesticide detection, with potential applications in environmental and agricultural sectors.

## Data availability

The data that support the findings of this study are available on request from the corresponding author.

## Author contributions

Sami Ullah Khan: performed the experiments and prepared initial draft. Muhammad Atif: structural studies, and Zulqurnain Ali: morphological studies. All authors have contributed to the interpretation of the results and written the manuscript. Waqas Khalid: supervision, project administration, manuscript preparation.

## Conflicts of interest

The authors declare that they have no known competing financial interests or personal relationships that could have appeared to influence the work reported in this paper.

## References

[cit1] Arias-Estévez M., López-Periago E., Martínez-Carballo E., Simal-Gándara J., Mejuto J.-C., García-Río L. (2008). The mobility and degradation of pesticides in soils and the pollution of groundwater resources. Agric., Ecosyst. Environ..

[cit2] Chen J., Peng Y., Li Y., Wang W., Wu J. (2011). A method for determining organophosphorus pesticide concentration based on near-infrared spectroscopy. Trans. ASABE.

[cit3] Pimentel D., Levitan L. (1986). Pesticides: amounts applied and amounts reaching pests. Bioscience.

[cit4] Bai Y., Zhou L., Wang J. (2006). Organophosphorus pesticide residues in market foods in Shaanxi area, China. Food Chem..

[cit5] Zhang Z. Y., Zhang C. Z., Liu X. J., Hong X. Y. (2006). Dynamics of pesticide residues in the autumn Chinese cabbage (Brassica chinensis L.) grown in open fields. Pest Manag. Sci..

[cit6] Liu W., Zhou Q., An J., Sun Y., Liu R. (2010). Variations in cadmium accumulation among Chinese cabbage cultivars and screening for Cd-safe cultivars. J. Hazard. Mater..

[cit7] Yadav M., Shukla A. K., Srivastva N., Upadhyay S. N., Dubey S. K. (2016). Utilization of microbial community potential for removal of chlorpyrifos: a review. Crit. Rev. Biotechnol..

[cit8] Mulla S. I. , Ameen F., Talwar M. P., Eqani S. A. M. A. S., Bharagava R. N., Saxena G., Tallur P. N. and Ninnekar H. Z., Organophosphate pesticides: impact on environment, toxicity, and their degradation, Bioremediation of Industrial Waste for Environmental Safety: Volume I: Industrial Waste and its Management, 2020, pp. 265–290

[cit9] Ma P., Wang L., Xu L., Li J., Zhang X., Chen H. (2020). Rapid quantitative determination of chlorpyrifos pesticide residues in tomatoes by surface-enhanced Raman spectroscopy. Eur. Food Res. Technol..

[cit10] Sinha S. N., Rao M. V. V., Vasudev K. (2012). Distribution of pesticides in different commonly used vegetables from Hyderabad, India. Food Res. Int..

[cit11] Hongsibsong S., Prapamontol T., Xu T., Hammock B. D., Wang H., Chen Z.-J., Xu Z.-L. (2020). Monitoring of the organophosphate pesticide chlorpyrifos in vegetable samples from local markets in Northern Thailand by developed immunoassay. Int. J. Environ. Res. Public Health.

[cit12] Yuan Y., Chen C., Zheng C., Wang X., Yang G., Wang Q., Zhang Z. (2014). Residue of chlorpyrifos and cypermethrin in vegetables and probabilistic exposure assessment for consumers in Zhejiang Province, China. Food Control.

[cit13] Rogers K. R., Wang Y., Mulchandani A., Mulchandani P., Chen W. (1999). Organophosphorus Hydrolase-Based Assay for Organophosphate Pesticides. Biotechnol. Prog..

[cit14] Albero B., Sánchez-Brunete C., Tadeo J. L. (2003). Determination of Organophosphorus Pesticides in Fruit Juices by Matrix Solid-Phase Dispersion and Gas Chromatography. J. Agric. Food Chem..

[cit15] Ciucu A. A., Negulescu C., Baldwin R. P. (2003). Detection of pesticides using an amperometric biosensor based on ferophthalocyanine chemically modified carbon paste electrode and immobilized bienzymatic system. Biosens. Bioelectron..

[cit16] Diagne M., Oturan N., Oturan M. A. (2007). Removal of methyl parathion from water by electrochemically generated Fenton's reagent. Chemosphere.

[cit17] Cho S.-K., Abd El-Aty A. M., Rahman M. M., Choi J.-H., Shim J.-H. (2014). Simultaneous multi-determination
and transfer of eight pesticide residues from green tea leaves to infusion using gas chromatography. Food Chem..

[cit18] Walorczyk S., Drożdżyński D., Kierzek R. (2015). Determination of pesticide residues in samples of green minor crops by gas chromatography and ultra performance liquid chromatography coupled to tandem quadrupole mass spectrometry. Talanta.

[cit19] Lu Y., Li X., Li W., Shen T., He Z., Zhang M., Zhang H., Sun Y., Liu F. (2021). Detection of chlorpyrifos and carbendazim residues in the cabbage using visible/near-infrared spectroscopy combined with chemometrics. Spectrochim. Acta, Part A.

[cit20] Jiang X., Li D., Xu X., Ying Y., Li Y., Ye Z., Wang J. (2008). Immunosensors for detection of pesticide residues. Biosens. Bioelectron..

[cit21] Torres C. M., Picó Y., Manes J. (1996). Determination of pesticide residues in fruit and vegetables. J. Chromatogr. A.

[cit22] Vrabelj T., Finšgar M. (2022). Recent progress in non-enzymatic electroanalytical detection of pesticides based on the use of functional nanomaterials as electrode modifiers. Biosensors.

[cit23] Hara T. O., Singh B. (2021). Electrochemical biosensors for detection of pesticides and heavy metal toxicants in water: recent trends and progress. ACS ES&T Water.

[cit24] Tun W. S. T., Saenchoopa A., Daduang S., Daduang J., Kulchat S., Patramanon R. (2023). Electrochemical biosensor based on cellulose nanofibers/graphene oxide and acetylcholinesterase for the detection of chlorpyrifos pesticide in water and fruit juice. RSC Adv..

[cit25] Wang H., Liang D., Xu Y., Liang X., Qiu X., Lin Z. (2021). A highly efficient photoelectrochemical sensor for detection of chlorpyrifos based on 2D/2D β-Bi2O3/g-C3N4 heterojunctions. Environ. Sci.: Nano.

[cit26] Kumaravel A., Murugananthan M. (2021). Electrochemical detection of fenitrothion using nanosilver/dodecane modified glassy carbon electrode. Sens. Actuators, B.

[cit27] Kumaravel A., Murugananthan M., Mangalam R., Jayakumar S. (2020). A novel, biocompatible and electrocatalytic stearic acid/nanosilver modified glassy carbon electrode for the sensing of paraoxon pesticide in food samples and commercial formulations. Food Chem..

[cit28] Khairy M., Ayoub H. A., Banks C. E. (2018). Non-enzymatic electrochemical platform for parathion pesticide sensing based on nanometer-sized nickel oxide modified screen-printed electrodes. Food Chem..

[cit29] Kumaravel A., Chandrasekaran M. (2010). A novel nanosilver/Nafion composite electrode for electrochemical sensing of methyl parathion and parathion. J. Electroanal. Chem..

[cit30] Kumaravel A., Chandrasekaran M. (2011). A biocompatible nano TiO2/Nafion composite modified glassy carbon electrode for the detection of fenitrothion. J. Electroanal. Chem..

[cit31] Nirmala J. N., Kumaravel A., Chandrasekaran M. (2010). Stearic acid modified glassy carbon electrode for electrochemical sensing of parathion and methyl parathion. J. Appl. Electrochem..

[cit32] Tian X., Liu L., Li Y., Yang C., Zhou Z., Nie Y., Wang Y. (2018). Nonenzymatic electrochemical sensor based on CuO-TiO2 for sensitive and selective detection of methyl parathion pesticide in ground water. Sens. Actuators, B.

[cit33] Tunesi M. M., Soomro R. A., Han X., Zhu Q., Wei Y., Xu B. (2021). Application of MXenes in environmental remediation technologies. Nano Convergence.

[cit34] Fu J., Tan X.-H., Li Y.-H., Song X.-J. (2016). A nanosilica/exfoliated graphene composite film-modified electrode for sensitive detection of methyl parathion. Chin. Chem. Lett..

[cit35] Song B., Cao W., Wang Y. (2016). A methyl parathion electrochemical sensor based on Nano-TiO2, graphene composite film modified electrode. Fullerenes, Nanotubes Carbon Nanostruct..

[cit36] Karimian N., Fakhri H., Amidi S., Hajian A., Arduini F., Bagheri H. (2019). A novel sensing layer based on metal–organic framework UiO-66 modified with TiO2–graphene oxide: application to rapid, sensitive and simultaneous determination of paraoxon and chlorpyrifos. New J. Chem..

[cit37] Abbasi M. A., Amin K. M., Ali M., Ali Z., Atif M., Ensinger W., Khalid W. (2022). Synergetic effect of adsorption-photocatalysis by GO−CeO2 nanocomposites for photodegradation of doxorubicin. J. Environ. Chem. Eng..

[cit38] Li T., Liu H. (2018). A simple synthesis method of nanocrystals CeO2 modified rGO composites as electrode materials for supercapacitors with long time cycling stability. Powder Technol..

[cit39] Sihotang H., Siburian R., Raja S. L., Supeno M., Simanjuntak C. (2018). New Route to Synthesize of Graphene Nano Sheets. Orient. J. Chem..

[cit40] Boehm H. P. (2002). Surface oxides on carbon and their analysis: a critical assessment. Carbon.

[cit41] Dong Y.-D., Shi Y., He Y.-L., Yang S.-R., Yu S.-Y., Xiong Z., Zhang H., Yao G., He C.-S., Lai B. (2023). Synthesis of Fe–Mn-Based Materials and Their Applications in Advanced Oxidation Processes for Wastewater Decontamination: A Review. Ind. Eng. Chem. Res..

[cit42] Nie X., Zhang R., Tang Z., Wang H., Deng P., Tang Y. (2020). Facile Fabrication of CeO2/Electrochemically Reduced Graphene Oxide Nanocomposites for Vanillin Detection in Commercial Food Products. Nanomaterials.

[cit43] Fischer J. , Barek J. and Wang J., New methods for electrochemical determination of pesticides (a review), Sensing in Electroanalysis, ed. K. Kalcher, R. Metelka, I. Švancara and K. Vytřas, 2012, vol. 7

[cit44] Bozal-Palabiyik B. , Dogan-Topal B., Ozkan S. A. and Uslu B., New Trends in Electrochemical Sensors Modified with Carbon Nanotubes and Graphene for Pharmaceutical Analysis, Novel Developments in Pharmaceutical and Biomedical Analysis, 2018, vol. 2, pp. 249–301

[cit45] Liu F.-m., Du Y.-q., Cheng Y.-m., Yin W., Hou C.-j., Huo D.-q., Chen C., Fa H.-b. (2016). A selective and sensitive sensor based on highly dispersed cobalt porphyrin-Co 3 O 4-graphene oxide nanocomposites for the detection of methyl parathion. J. Solid State Electrochem..

[cit46] Karimi-Maleh H., Darabi R., Baghayeri M., Karimi F., Fu L., Rouhi J., Niculina D. E., Gündüz E. S., Dragoi E. N. (2023). Recent developments in carbon nanomaterials-based electrochemical sensors for methyl parathion detection. J. Food Meas. Char..

[cit47] Alshareef F. M., Orif M. I., Al-Harbi E. A., El-Shahawi M. S. (2021). A highly sensitive electrochemical sensor based on electrocatalytic reduction effect of Cu2+ on trace determination of malathion in soil and other complex matrices. Int. J. Electrochem..

[cit48] Fu X.-C., Zhang J., Tao Y.-Y., Wu J., Xie C.-G., Kong L.-T. (2015). Three-dimensional mono-6-thio-β-cyclodextrin covalently functionalized gold nanoparticle/single-wall carbon nanotube hybrids for highly sensitive and selective electrochemical determination of methyl parathion. Electrochim. Acta.

[cit49] Jahromi M. N., Tayadon F., Bagheri H. (2020). A new electrochemical sensor based on an Au-Pd/reduced graphene oxide nanocomposite for determination of Parathion. Int. J. Environ. Anal. Chem..

[cit50] Kumaravel A., Chandrasekaran M. (2015). Electrochemical determination of chlorpyrifos on
a nano-TiO2/cellulose acetate composite modified glassy carbon electrode. J. Agric. Food Chem..

[cit51] Tun W. S. T., Saenchoopa A., Daduang S., Daduang J., Kulchat S., Patramanon R. (2023). Electrochemical biosensor based on cellulose nanofibers/graphene oxide and acetylcholinesterase for the detection of chlorpyrifos pesticide in water and fruit juice. RSC Adv..

[cit52] Chauhan N., Narang J., Jain U. (2016). Amperometric acetylcholinesterase biosensor for pesticides monitoring utilising iron oxide nanoparticles and poly(indole-5-carboxylic acid). J. Exp. Nanosci..

[cit53] Mogha N. K., Sahu V., Sharma M., Sharma R. K., Masram D. T. (2016). Biocompatible ZrO2-reduced graphene oxide immobilized AChE biosensor for chlorpyrifos detection. Mater. Des..

[cit54] Xu G., Huo D., Hou C., Zhao Y., Bao J., Yang M., Fa H. (2018). A regenerative and selective electrochemical aptasensor based on copper oxide nanoflowers-single walled carbon nanotubes nanocomposite for chlorpyrifos detection. Talanta.

[cit55] Guler M., Turkoglu V., Basi Z. (2017). Determination of malation, methidathion, and chlorpyrifos ethyl pesticides using acetylcholinesterase biosensor based on Nafion/Ag@rGO-NH2 nanocomposites. Electrochim. Acta.

[cit56] Talan A., Mishra A., Eremin S. A., Narang J., Kumar A., Gandhi S. (2018). Ultrasensitive electrochemical immuno-sensing platform based on gold nanoparticles triggering chlorpyrifos detection in fruits and vegetables. Biosens. Bioelectron..

